# The European Conference on Computational Biology 2002

**DOI:** 10.1002/cfg.256

**Published:** 2003-02

**Authors:** Clare Sansom

**Affiliations:** School of Crystallography Birkbeck College University of London Malet Street London WC1E 7HX UK


**Comparative and Functional Genomics**
*Comp Funct Genom* 2003; **4**: 20–24.
Published online in Wiley InterScience (www.interscience.wiley.com). **DOI:** 10.1002/cfg.256

**Feature**

### Meeting Review: The European Conference on Computational Biology 2002

Saarbrücken, Germany, 6–9 October 2002

Clare Sansom*
*School of Crystallography, Birkbeck College, University of London, Malet Street, London WC1E 7HX, UK*

**Correspondence to:
Clare Sansom, School of Crystallography, Birkbeck College, University of London, Malet Street, London WC1E 7HX, UK.
E-mail: c.sansom@mail.cryst.bbk.ac.uk*

Received: 6 December 2002
Accepted: 6 December 2002

### Introduction

With this conference, styled 'ECCB 2002' and held in Saarbrücken, Germany, on 6–9 October, the bioinformatics community in Europe entered a new era. It was the first computational biology conference to have a specifically pan-European remit. In future, a conference in the ECCB series will be held annually. Each meeting will be hosted by a different country or region, in conjunction with that country's national bioinformatics conference.

This first meeting was held with the annual German Conference on Bioinformatics (GCB). It followed a similar format to the ISMB and RECOMB conference series, with papers for oral presentation (other than those by the invited keynote speakers) chosen by peer review and published in a special issue of the journal *Bioinformatics* [1]. Twenty-six papers were chosen from 83 submissions, and about 190 posters were presented: encouraging figures for a conference series that has yet to become established.

### Networks and expression data

The first keynote lecture was given by **Scott Patterson (Celera Genomics, Rockville, USA).** He described the industrial-scale facilities for proteomics — which he defined as the identification of differentially expressed proteins and measurement of protein levels — that have been set up at Celera. Although the established combination of 2D-PAGE and mass spectroscopy is still the technique that can separate proteins at the highest resolution, other techniques, including protein chips, are improving rapidly. Bioinformatics challenges posed by proteomics include data reduction, analysis and visualization: the first of these is particularly important, since a large mass spectrometry facility alone can produce as much as 30 Gb of data each day. Researchers at Celera are using these facilities to detect proteins that are differentially expressed in pancreatic cancer cells. There is a great need for reliable markers for this disease, which has an exceptionally poor prognosis, largely because it is usually diagnosed at a late stage.

**Thomas Schlitt (EMBL-EBI, Hinxton, UK)** described an analysis of gene dependency networks, derived from a microarray analysis of 248 single gene deletion mutants of yeast. Nodes in the network represent genes, with an arrow linking each gene deleted to those affected by its deletion (also presented by Rung at the CCP11 group meeting, Manchester 2002 [8]). Schlitt and colleagues

Copyright © 2003 John Wiley & Sons, Ltd.

show that the network is similar to scale-free networks and consists of only one major component. Genes involved in the same processes are often located close together in the network, e.g. many nearest neighbours of genes involved in mating are involved in pheromone responses.

Gene regulation in eukaryotes is controlled by transcription factors, through interactions with DNA that are complex and still poorly understood. **Kimmo Palin (University of Helsinki, Finland)** and colleagues have defined gene sets known to be linked via disruption (in a similar manner to Schlitt) and other sets linking transcription factors and genes that contain the promoter regions that they bind to. There will often, but by no means always, be a correlation between the set of genes disrupted by the knockout of a given transcription factor and the set containing binding sites for that transcription factor. Palin and colleagues tested this hypothesis using experimental gene expression data [4] and known transcription factor binding motifs [6], and found that 20 out of 37 binding motifs correlated with the disruption experiments. Some of the strongest correlations, however, had no obvious biological explanation.

### Evolution and phylogeny

Now that many complete bacterial genome sequences are available in the public domain, our view of the evolution of prokaryotes is changing rapidly. **Siv Andersson (University of Uppsala, Sweden)** has compared the genomes of closely related pairs of intracellular pathogens and symbionts from the genera *Rickettsia, Bartonella* and *Buchnera*. Typically, differences between related pathogenic bacteria with, for instance, different routes of infection or host ranges, are concentrated in repeats and pathogenicity islands, e.g. in the genus *Rickettsia* the typhus bacterium *Rickettsia typhi* is effectively a subset of the larger *R. conorii*. About 80% of the genes that are found in *R. conorii* but not in *R. typhi* are, in fact, orphan genes — genes that are unique to that organism. Andersson found that these 'ORFans' were often significantly shorter than those *R. conorii* genes with orthologues. She has proposed the theory that many of these genes may be non-functional fragments of ancestral genes. Defining criteria for the identification of horizontal gene transfer events can present major difficulties. A case study of the *Buchnera* phylome suggests that atypical phylogenetic trees cannot be taken as indications of horizontal gene transfer events.

The challenge of understanding the torrent of genomic information now entering the public domain, with about 100 complete genomes now available, also motivates **Laurent Duret's (Université Claude Bernard, Lyon, France)** studies in functional genomics [2]. Large-scale functional genomics studies of yeast have revealed that a surprisingly high percentage of yeast genes — possibly as many as 50% — appear to have little effect on the organism's function. This result may be due to inappropriate testing, functional redundancy or the fact that those genes make only a marginal contribution to fitness. Duret believes that the third explanation is most likely in many cases. He is analysing the patterns of synonymous codon usage in genes with different levels of expression, and has found a correlation between optimal codon usage and high gene expression in *Caenorhabditis elegans, Drosophila melanogaster* and *Arabidopsis thaliana*, but not in human. This type of comparative analysis may be used to detect genomic features under weak selective pressure.

**Olivier Elemento (Université Montpellier, France)** described DTSCORE, a fast algorithm for reconstructing tandem duplication trees. These trees describe the order in which two or more duplications of the same short DNA fragment occurred in, for example, microsatellites or minisatellites. This is a complex process; DTSCORE is based on the ADDTREE algorithm [9] but its time complexity has been reduced from $O(n^5)$ to $O(n^4)$. Tests with real genomic data have shown that this algorithm returns the correct tree more often than comparable methods, including neighbour joining trees. It was most accurate when the number of repeat copies was high.

### Gene and protein function

The term 'metabolomics' can now be applied to the study of the small molecule complement of cell types, by analogy with proteomics. Any change in gene expression brought about by, for example, environmental or developmental change will affect the metabolome; genotypes may also

Copyright © 2003 John Wiley & Sons, Ltd. 

be distinguished by the metabolites produced in their cells. **Janet Taylor (University of Wales, Aberystwyth, UK)** has used neural networks to distinguish between the metabolite patterns of *A. thaliana* genotypes and crosses. She proved that it was possible, not only to distinguish between the pure *A. thaliana* background lines Col0 and C24, but to distinguish between the two forms of first-generation progeny (Col0 × C24 and C24 × Col0), which differ only in their maternally inherited mitochondrial and chloroplast genomes. Malic acid and citrate were found to be the most important metabolites for discriminating between the background lines, and glucose and fructose were most important for distinguishing between the crosses. Mitochondria and chloroplasts play a key role in the synthesis and catabolism of these sugars. It would be useful to link these analyses back to studies of the proteome, but this is currently limited by the lack of any common and systematic nomenclature or ontology for metabolites.

**Jan Freudenberg (Universität Bonn, Germany)** and his colleagues are using an index of disease phenotypes, as described in the OMIM database, to predict genes linked with disease. They have proposed that diseases with similar phenotypes are likely to be caused by genes of similar molecular function. Freudenberg assigned keywords that describe disease attributes, including etiology, affected tissue, and age of onset; e.g. colorectal cancer is described as a neoplastic disease of late adulthood affecting the gastro-intestinal tract, whereas muscular dystrophy is a degenerative disease of childhood affecting the muscles. Potential disease genes were scored according to their similarity to genes that are linked with diseases with similar attributes. Using the technique of leave-one-out cross-validation, the approach was shown to have variable success in predicting disease genes. Predictions for genes underlying eye diseases scored higher on average than those for cardiac genes, possibly because many eye diseases are monogenic and well understood.

Analysis of the interaction between ligands and protein binding sites is an important part of many drug discovery programs, yet it is still difficult to detect the optimum position for ligand binding. **Douglas Brutlag (Stanford University, California, USA)** gave a keynote lecture describing a novel method of ligand docking. The technique of stochastic roadmap simulation (SRS; not to be confused with the Sequence Retrieval System that is familiar to most bioinformaticians) maps a complex conformational problem in three-dimensional space, with many degrees of freedom, into a much simpler problem in many dimensions. Calculating a stochastic roadmap is equivalent to calculating all possible Monte Carlo paths through a simulation at once; many can be ruled out automatically because of steric clashes between atoms. Brutlag's colleague, **M. Serkan Apadyin**, described an application of SRS to measuring the effect of active site mutations on ligand binding. He measured the time (in Monte Carlo time steps) it took for a ligand to escape from the vicinity of its binding site, and compared this 'escape time' in wild-type and mutant enzymes. He was able to correlate differences in escape times in several mutants of lactate dehydrogenase with changes in expected binding affinity that have been predicted from the chemical properties of the mutated residues (see e.g. [10]).

### Protein analysis and classification

Many evolutionary relationships between proteins cannot be easily detected by sequence analysis because the percentage sequence identity between the proteins is low: they are hidden in the so-called 'twilight zone'. **Alexander Schliep (Max Planck Institute for Molecular Genetics, Berlin)** and colleagues have developed ProClust, a graph-based algorithm for clustering similar protein sequences. Scores were scaled to account for differences in the lengths of the sequences. The initial method was quite conservative, sometimes dividing a SCOP superfamily into several small clusters; the results were improved by the addition of a step in which clusters were merged using Hidden Markov Models. The combined method was more sensitive than PSI-Blast at the same specificity, although it was also much more computationally intensive. This advantage over PSI-BLAST was particularly clear in the analysis of multidomain proteins.

It is well known that the rate at which sequences enter the databases far outstrips that at which those sequences can be fully annotated, leaving some database entries incomplete. As automatic annotation methods become more accurate, this gap is slowly being closed. **Ana Bazzan (Instituto de Informática, Porto Alegre, Brazil)** has developed

Copyright © 2003 John Wiley & Sons, Ltd. 

an automatic annotation procedure that is specific for protein sequences from pathogenic bacteria of the family Mycoplasmataceae. This family includes *Mycoplasma hyopneumoniae*, which causes chronic respiratory disease and is enzootic in the pig population of Brazil. Bazzan and colleagues have used partly annotated data gathered from genome projects on organisms of that family to generate a series of rules for the addition of SWISS-PROT keywords to previously unannotated sequences. The method is based on the algorithm C4.5 [7]; a typical generated rule might be, 'If the protein has the InterPro classification IPR000158, then it shall have the keyword "Cell Division"'. If all tests for a given keyword fail, the protein is not annotated with that keyword. They eventually aim to extend the method to other types of annotation within SWISS-PROT.

**Sarah Teichmann (MRC Laboratory of Molecular Biology, Cambridge, UK)** presented a summary of her research on protein-protein interactions. Interactions between protein domains are central to the functioning of organisms: they occur in multidomain proteins, in stable interactions between domains (e.g. those between the four globin chains in haemoglobin) and in transient interactions (e.g. that between a ligand and its receptor). She found less sequence divergence on average between orthologous proteins involved in stable interactions than in monomeric proteins, with those involved in transient interactions falling in between. This could be attributed to the fact that the stable interactions bury larger surface areas when they form. Following on from Andersson's speculations about problems with horizontal gene transfer, she suggested that the most accurate phylogenetic trees might be calculated by using proteins involved in stable complexes, which are both more conserved and less likely to be horizontally transferred. It is known that proteins in stable complexes also tend to be co-expressed, and Teichmann has shown that this co-regulation is frequently conserved across distantly related eukaryotes.

### Genome analysis

**Christoph Dieterich**, from Martin Vingron's group in the **Max-Planck Institute for Molecular Genetics, Berlin, Germany**, described a method for predicting regulatory sequences of DNA based on synteny between the genomes of man and mouse. Many workers have predicted that those regions of non-coding DNA that are conserved between these two well-studied mammalian genomes are likely to contain transcription factor binding sites (for review, see [3]). Dieterich and co-workers investigated DNA regions upstream of orthologous gene pairs from the human and mouse genomes. A variant of the Waterman–Eggert algorithm [5] was employed to identify local suboptimal sequence alignments that were statistically significant. These alignments preferentially cover experimentally verified promoter sequences.

**Pierre Nicodeme (CNRS-EVRY, Evry, France)** described a collaboration with the Vingron group in Berlin to study residue patterns in whole proteomes. Many of the functional motifs in the Prosite database are only a few amino acids in length and random occurrences of such patterns are bound to occur quite often in sequences the length of whole proteomes. Using Bernoulli statistics, Nicodeme calculated the number of times known motifs would be expected to occur in complete proteomes if the patterns were indeed random and compared this to the number found by sequence analysis. He found that patterns were, in general, overrepresented in genomes where they were known to occur as functional motifs, e.g. the common 'C2H2' zinc finger pattern was found 45 times as often as it was expected in the *Drosophila* proteome, but was not significantly overrepresented in any of the prokaryotic proteomes tested. Prokaryotes do not employ zinc fingers as DNA binding motifs. Other patterns, such as the Arg–Gly–Asp cell adhesion motif, tended to be underrepresented in proteins where it was not expected to be functional; not unsurprising, since chance occurrence of an adhesion motif might well have unwanted effects.

The second European conference on computational biology will be held in Paris on 27–30 September 2003, in conjunction with the annual French computational biology conference, JOBIM. If it lives up to the standard set by this first ECCB, it will be well worth a place in any bioinformatician's schedule.

### References

1. 2002. *Bioinformatics* **18**(suppl 2).
2. Duret L. 2002. Evolution of synonymous codon usage in metazoans. *Curr Opin Genet Dev* **12**: 640–649.

Copyright © 2003 John Wiley & Sons, Ltd. 

3. Hardison R. 2000. Conserved non-coding sequences are reliable guides to regulatory elements. *Trends Genet* **16**: 369–372.
4. Hughes TR, Marton MJ, Jones AR, *et al*. 2000. Functional discovery via a compendium of expression profiles. *Cell* **102**: 109–126.
5. Huang X, Miller W. 1991. *Adv Appl Math* **12**: 337–357.
6. Pilpel Y, Sudarsanam P, Church GM. 2001. Identifying regulatory networks by combinatorial analysis of promoter elements. *Nature Genet* **29**: 153–159.
7. Ross Quinlan's home page: **http://www.cse.unsw.edu.au/~quinlan/**
8. Sansom CE. 2002. Meeting Review: CCP11 group meeting — towards the functional analysis of microarrays. *Comp Funct Genom* **3**(5): 451–454.
9. Sattath S, Tversky A. 1977. *Psychometrika* **42**: 319–345.
10. Wilks HM, Hart KW, Feeney R, *et al*. 1988. A specific, highly active malate dehydrogenase by redesign of a lactate dehydrogenase framework. *Science* **242**: 1541–1544.

---

The Meeting Reviews of *Comparative and Functional Genomics* aim to present a commentary on the topical issues in genomics studies presented at a conference. The Meeting Reviews are invited; they represent personal critical analyses of the current reports and aim at providing implications for future genomics studies.

---

Copyright © 2003 John Wiley & Sons, Ltd. 

## References

[PDF_00367] Duret Laurent (2002). Evolution of synonymous codon usage in metazoans.. Curr Opin Genet Dev.

[PDF_00371] Hardison R. C. (2000). Conserved noncoding sequences are reliable guides to regulatory elements.. Trends Genet.

[PDF_00374] Hughes T. R., Marton M. J., Jones A. R., Roberts C. J., Stoughton R., Armour C. D., Bennett H. A., Coffey E., Dai H., He Y. D. (2000). Functional discovery via a compendium of expression profiles.. Cell.

[PDF_00378] Pilpel Y., Sudarsanam P., Church G. M. (2001). Identifying regulatory networks by combinatorial analysis of promoter elements.. Nat Genet.

[PDF_00383] Sansom Clare (2002). CCP11 group meeting - towards the functional analysis of microarrays.. Comp Funct Genomics.

[PDF_00387] Wilks H. M., Hart K. W., Feeney R., Dunn C. R., Muirhead H., Chia W. N., Barstow D. A., Atkinson T., Clarke A. R., Holbrook J. J. (1988). A specific, highly active malate dehydrogenase by redesign of a lactate dehydrogenase framework.. Science.

